# Sexual Dimorphism in Melanin Pigmentation, Feather Coloration and Its Heritability in the Barn Swallow (*Hirundo rustica*)

**DOI:** 10.1371/journal.pone.0058024

**Published:** 2013-02-28

**Authors:** Nicola Saino, Maria Romano, Diego Rubolini, Celine Teplitsky, Roberto Ambrosini, Manuela Caprioli, Luca Canova, Kazumasa Wakamatsu

**Affiliations:** 1 Department of Biosciences, University of Milan, Milan, Italy; 2 Départment Ecologie et Gestion de la Biodiversité UMR 7204, CNRS/MNHN/UPMC, Paris, France; 3 Department of Biothecnology and Biosciences, University of Milano-Bicocca, Milano, Italy; 4 Dipartimento di Biologia e Biotecnologie, Università degli Studi di Pavia, Italy; 5 Department of Chemistry, Fujita Health University School of Health Sciences, Toyoake, Aichi, Japan; University of Lausanne, Switzerland

## Abstract

Melanin is the main pigment in animal coloration and considerable variation in the concentrations of the two melanin forms (pheo- and eumlanin) in pigmented tissues exists among populations and individuals. Melanin-based coloration is receiving increasing attention particularly in socio-sexual communication contexts because the melanocortin system has been hypothesized to provide a mechanistic basis for covariation between coloration and fitness traits. However, with few notable exceptions, little detailed information is available on inter-individual and inter-population variation in melanin pigmentation and on its environmental, genetic and ontogenetic components. Here, we investigate melanin-based coloration in an Italian population of a passerine bird, the barn swallow (*Hirundo rustica rustica*), its sex- and age-related variation, and heritability. The concentrations of eu- and pheomelanin in the throat (brown) and belly (white-to-brownish) feathers differed between sexes but not according to age. The relative concentration of either melanin (Pheo:Eu) differed between sexes in throat but not in belly feathers, and the concentrations in males compared to females were larger in belly than in throat feathers. There were weak correlations between the concentrations of melanins within as well as among plumage regions. Coloration of belly feathers was predicted by the concentration of both melanins whereas coloration of throat feathers was only predicted by pheomelanin in females. In addition, Pheo:Eu predicted coloration of throat feathers in females and that of belly feathers in males. Finally, we found high heritability of color of throat feathers. Melanization was found to differ from that recorded in *Hirundo rustica rustica* from Scotland or from *H. r. erythrogaster* from North America. Hence, present results show that pigmentation strategies vary in a complex manner according to sex and plumage region, and also among geographical populations, potentially reflecting adaptation to different natural and sexual selection regimes, and that some coloration components seem to be highly heritable.

## Introduction

Organisms vary in coloration of their body surfaces that are exposed to the external environment. Due to the diverse and potentially contrasting natural and sexual selection pressures that act on coloration, the functional analysis of such variation occurring both at the intra- and inter-specific level is complex [1]–[5]. Coloration can affect body thermal balance via differential reflection of solar radiation and is crucial to crypsis and mimicry of both prey and predators [6]–[9]. In addition, socio-sexual communication and species recognition are often mediated by colorful signals [2], [4], [10], [11]. Yet, the possibility that individual and inter-population variation in coloration may in some instances be an essentially neutral side-effect of geographical variation in ecology or physiology cannot be dismissed a priori.

From a different perspective, the analysis of color variation should rely on the dissection of the mechanisms and constraints that cause it, and on the costs-to-benefits balance of *ex novo* production or allocation of dietary pigments to the production of traits under natural and sexual selection. For example, condition-dependent expression of melanin- or carotenoid-based coloration may have to be balanced against costs that may be paid in diverse currencies ([11]–[15], but see [16], [17]), and it has thus been proposed that such trade-offs underpin the reliability of coloration in signaling individual quality or alternative life-history strategies under frequency-dependent or balancing selection.

Aside from highly specialized neural or endocrine control of dynamic structural or pigmentary coloration (e.g. [18], [19]), integumental coloration is generally determined by persistent, relatively static accumulation of pigments that can be either exogenous or endogenously produced from their dietary precursors. Melanin is the most common endogenous pigment. Besides being involved in thermoregulation, camouflage and signaling functions (see also above), melanin that is accumulated in the integument also plays roles that, depending on the taxon, range from protection from abrasion and solar radiation, to defense from oxidative stress and pathogens (see [20]).

Melanogenesis starts from the biosynthesis of dopaquinone from L-tyrosine in a reaction catalyzed by tyrosinase, and proceeds through sequential oxidation and decarboxylation intermediate steps most often leading to the production of both melanin forms: pheomelanin, which is responsible for yellow to reddish-brown hues, and eumelanin, which is responsible for brown to black hues [21]–[25]. However, the specific profile of such ‘mixed melanogenesis’, in terms of proportion of eu- and pheomelanin, can vary among tissues as well as among individuals. Thus, melanic hues may result from a combination of variation in absolute and relative concentration of either melanin [26]–[28]. Moreover, ontogenetic variation can occur, with an age-related change in absolute as well as relative production of either melanin form [29], [30].

Importantly, it has been hypothesized that a key role in the evolution of melanic coloration particularly in communication contexts is played by the melanocortin system (see [31]). In vertebrates, the *POMC* gene encoding melanocortins link to the melanocortin-1 receptor (MC1R), triggering melanogenesis, but also to other receptors involved in the regulation of major fitness traits such as sexual behavior, immunity, energy homeostasis and response to stress via the HPA axis [31]. Such pleiotropic effects of *POMC* may provide a mechanistic basis to interpret the observed covariation between melanin-based coloration and diverse fitness components, including sexual behavior, aggressiveness, stress response as mediated by the HPA axis, immunity and energy homeostasis (reviewed in [3], [20], [31]; e.g. [32]).

Birds constitute no exception to the widespread occurrence of melanic pigmentation as coloration of feathers and other exposed surfaces (e.g. bill) is mainly based on ‘mixed’ melanins [33]. Despite the functional importance of melanic coloration [2] and its obvious patterns of habitat-dependent variation (e.g. according to humidity gradients, as generalized in Gloger’s rule), only relatively few studies have investigated the biochemical composition of melanins in the feathers and its sex- and age- or condition-dependent variation [29], [34], [35]. Very few, though exceptionally detailed studies of the phenotypic covariation between melanic pigmentation and fitness traits putatively under the pleiotropic influence of the same genetic machinery exist [3], [31]. In addition, there is a dearth of studies on the extent of genetic and environmental components of variation in melanism at the within- as well as among-populations levels, although tight genetic control has often been invoked (e.g. [1], [3], [13], [14], [36]–[40]; see also [41]). Indeed, while there is plenty of evidence of parent-offspring resemblance in melanin-based coloration, only very few studies have been able to partition genetic and environmental causes of such resemblance ([42], see [3]). Moreover, there is both correlative and experimental evidence that nutritional condition including availability of cysteine at the level of melanosomes [28], [43], variation in food abundance [14], parasitism [13] and individual variation in androgens profile [44] can affect melanogenesis.

In the present study we thus first describe the eu- and pheomelanin content of the feathers from two plumage regions of barn swallows (*Hirundo rustica rustica*) from a population breeding in northern Italy and investigate its variation in relation to sex and age. Throat and/or belly feathers have been shown to exhibit sex differences in a European population of *H. r. rustica* and in the North American subspecies *Hirundo rustica erythrogaster* [34], [35], [45]. Throat and belly feathers were chosen as representatives of the white-brownish breast, belly and axillary plumage regions and, respectively, of the brown head regions (throat and front). We then analyze the covariation between feather coloration and absolute or relative concentration of either melanin form. Because we were interested in any potential role of coloration as a socio-sexual signal, we quantified coloration from a bird’s visual system perspective, i.e. by taking visual spectral sensitivities of passerine birds into account. This is important because visual system-independent spectral color metrics may confound the appreciation of coloration information content as perceived by birds [46]–[48]. Finally, we analyze heritability in coloration by exploiting frequent occurrence of extra-pair paternity to partition any environmental component due to shared nest environment from the genetic effects on resemblance between offspring and parents when they eventually became adults. In the present study, we will not specifically deal with sex dichromatism, which will be at the focus of a companion paper based on a very large sample of birds from several years. The present study will serve as a basis for future studies on the covariation between melanin-based coloration and fitness traits in our model species.

## Materials and Methods

### Study Species

The barn swallow is a small (ca. 20 g), socially monogamous, colonial, passerine bird. Its breeding range encompasses the entire Holoarctic Region while the wintering quarters of migratory populations are located in the tropics. Seven subspecies are currently recognized which differ in several aspects of behavior, morphology and coloration [49]–[51]. Populations breeding in Europe undergo a single annual molt of wing and tail feathers during the wintering period in sub-Saharan Africa. Contour body feathers undergo a post-breeding, pre-migratory partial molt and then a second molt episode during wintering [52]. Male traits that have been shown to be currently under sexual selection are, depending on the population, length and symmetry of the outermost tail feathers, coloration of breast and belly feathers, and song [49]–[51], [53]–[57].

### Field Procedures

The analysis of feather pigmentation and of its association with coloration was carried out on feathers sampled during spring 2012 in the breeding colonies located in four farms in our study area near Milano (Northern Italy) where we had been studying swallows since at least three years. In May we captured the breeding adults and marked them with individually numbered metal rings and colored plastic rings. We plucked approximately 30 feathers from the center of the brown throat patch and 5 feathers from the belly, and stored them in plastic bags in a dark place at room temperature until melanin and color analysis in July 2012. Feathers were plucked from the same position in all individuals. Sex was identified based on morphology and socio-sexual behavior observed at the nest [49], and later confirmed by standard molecular techniques using a small blood sample in case of uncertain assignment based on morphology [58].

### Age Determination

The age of adult barn swallows cannot be assigned after the first complete molt of the plumage, that occurs during the first winter in Africa. Barn swallows have extremely high breeding philopatry in the two study areas in northern Italy where we have been working since several years as well as in other European regions (southern Switzerland, C. Scandolara, pers. comm.; Denmark, [49]), implying that an individual that has bred in a particular farm only very rarely moves to another farm to breed in the following years. Conversely, natal philopatry is low and most offspring move to a different colony, located at variable distance from their original one, to breed. Because adults normally spend the night inside the buildings where they breed, they can be efficiently captured by placing mist nets at the exits of the buildings before dawn. As a result of repeated capture sessions throughout the breeding season (April-July) extremely few adults may escape capture. Breeding philopatry and high capture efficiency imply that individuals that are captured in a particular year and colony but had not been captured in that colony in the previous year can be confidently assumed to be yearlings, thus allowing to accurately estimate their age in subsequent years (see [59]–[61]). For the purposes of the present study we classified the individuals as either yearlings (i.e. individuals hatched approximately one year before the study) or older individuals. This classification should capture a very large proportion of the age-related variation in pigment concentration at the population level, given the low annual survival rate (ca. 0.3–0.4) of adult barn swallows, which implies that individuals older than two years account for ca. 15% or less of the population.

### Feather Color Measurements

**Figure 1:**
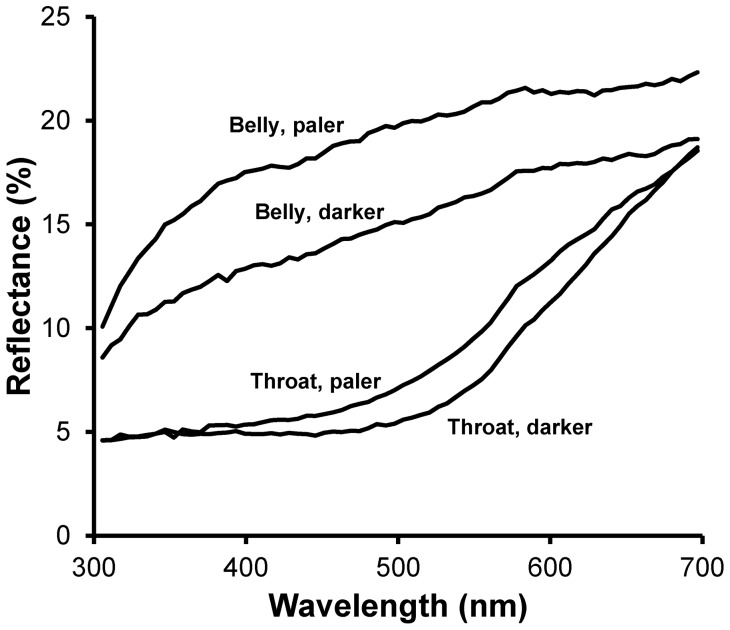
Reflectance spectra of belly or throat feathers with relative pale or dark color.

We quantified feather reflectance by means of spectrophotometric measurements using an Avantes DH-2000 spectrometer in a dark chamber (Fig. 1). Illumination was provided by a combined deuterium-tungsten halogen light source. Two repeated measurements were obtained from three overlain throat feathers or one belly feather. Reflectance of the samples was always referred to white and black standards. The illuminated field covered an area of 2.5 mm^2^ centered approximately 1.5 mm from the distal end of the throat feathers (i.e. in the terminal brown part of the feather) and 2.5 mm from the distal end of the belly feather (i.e. in a white-to-brownish region). Reflectance spectra were processed to quantify coloration using the tetrachromatic color space model [62] using TetraColorSpace program (Version 1a; [47]) run in MATLAB 7 (MathWorks, Natick, MA). This approach has the advantage of incorporating information on both plumage reflectance spectra and bird cone sensitivity functions. This allows to estimate the relative stimulation of the retinal cones and thus to better model the color perceived by birds. As this model has been recently adopted in a number of studies of tetrachromatic vertebrates (e.g. [47], [63]), we will only briefly describe the rationale of the method here. According to Goldsmith and co-workers [62], the idealized stimulus Q_I_ of each retinal cone type by the reflectance of a color patch can be estimated as:
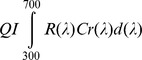
where R(λ) is the color reflectance spectrum, C_r_(λ) is the spectral sensitivity function of cone type r. We assumed UVS cone type-retina and used spectral sensitivity of the blue tit (*Cyanistes caeruleus*) because this is the species more phylogenetically close to the barn swallow for which spectral sensitivity information is implemented in TetraColorSpace program. However, correlation analysis of θ and φ color components (see below) obtained by assuming blue tit or, respectively average bird UVS spectral sensitivities disclosed an extremely high association (details not shown). This implies that the results were robust to the effect of the particular spectral sensitivity profile chosen among those typical of UVS birds. Idealized stimulation of the four cones were normalized to a sum of 1, so that each color patch can be described in the tetrahedral color space by a vector of {uv, s, m, l} values representing the relative stimulations of the ultraviolet-, short-wavelength-, medium-wavelength-, and long-wavelength-sensitive cones, respectively. Each color vector in the tetrahedral color space is then transformed to Cartesian coordinates that are subsequently converted into the spherical coordinates θ, φ, and r (see [47], [63]). θ and φ that we used here to quantify hue roughly represent the red-green-blue (θ) or the ultraviolet (φ) components of hue, while r is a measure of color saturation (or chroma). In the range of colors of barn swallow throat and belly feathers, increasing θ values indicate paler, whitish coloration. No verbal description can be provided for variation in φ because this mainly reflects ultraviolet hue components which cannot be sensed by the human eye. Because the color space is a tetrahedron and not a sphere, different hues vary in their maximum potential chroma (r_max_) [47]. Thus, in the analyses we used the ‘achieved chroma’, computed as rA = r/r_max_.

Repeatability [64] of the coloration variables as estimated by measuring twice the same feather(s) at both plumage regions was always larger than 0.73 (n = 45 individuals for both plumage regions). Among-feathers repeatability estimated by measuring two different feathers from the same region was also larger than 0.74 for all variables (n = 10 individuals for both plumage regions).

### Melanin Analyses

The microanalytical methods we used to quantify the amounts of eumelanin and pheomelanin (see [65]) are based on the formation of specific degradation products: pyrrole-2,3,5-tricarboxylic acid (PTCA) by alkaline H_2_O_2_ oxidation (acidic KMnO_4_ oxidation in the original method; [65]) of eumelanin and 4-amino-3-hydroxyphenylalanine (4-AHP) by reductive hydrolysis of pheomelanin with hydriodic acid (HI). After trimming, brown barbs of the throat feathers and white-brownish barbs of belly feathers were homogeneized at a concentration of 10 mg/mL in water. The methods for alkaline H_2_O_2_ oxidation measure of eumelanin are reported in [66]). Sample homogenates (100 µL) were taken in a 10 ml screw-capped conical test tube, to which 375 µL 1 mol/L K_2_CO_3_ and 25 µL 30% H_2_O_2_ (final concentration: 1.5%) were added. The mixture was mixed vigorously at 25°C ±1°C for 20 hr. The residual H_2_O_2_ was decomposed by adding 50 µL 10% Na_2_SO_3_ and the mixture was then acidified with 140 µL 6 mol/L HCl. After vortex-mixing, the reaction mixture was centrifuged at 4,000 g for 1 min, and an aliquot (80 µL) of the supernatant was directly injected into the HPLC system.

The methods for HI reductive hydrolysis to measure pheomelanin (4-AHP) are described in [24], [65]. Sample homogenates (100 µL) were taken in a 10 ml screw-capped conical test tube, to which 20 µL 50% H_3_PO_2_ and 500 µL 57% HI were added. The tube was heated at 130°C for 20 hr, after which the mixture was cooled. An aliquot (100 µL) of each hydrolysate was transferred to a test tube and evaporated to dryness using a vacuum pump connected to a dry ice-cooled vacuum trap and two filter flasks containing NaOH pellets. The residue was dissolved in 200 µL 0.1 mol/L HCl. An aliquot (10–20 µL) of each solution was analyzed on the HPLC system.

H_2_O_2_ oxidation products were analyzed with the HPLC system consisting of a JASCO 880-PU liquid chromatograph (JASCO Co., Tokyo, Japan), a Shiseido C_18_ column (Shiseido Capcell Pak MG; 4.6×250 mm; 5 µm particle size) and a JASCO UV detector. The mobile phase was 0.1 mol/L potassium phosphate buffer (pH 2.1) : methanol, 99∶1 (v/v). Analyses were performed at 45°C at a flow rate of 0.7 mL/min. Absorbance of the eluent was monitored at 269 nm. A standard solution (80 µL) containing 1 µg of PTCA (pyrrole-2,3,5-tricarboxylic acid) in 1 mL water was injected every 10 samples. HI reductive hydrolysis products were analyzed with an HPLC system consisting of a JASCO 880-PU liquid chromatograph, a JASCO C_18_ column (JASCO Catecholpak; 4.6×150 mm; 7 µm particle size) and an EICOM ECD-300 electrochemical detector. The mobile phase used for analysis of 4-AHP was 0.1 mol/L sodium citrate buffer, pH 3.0, containing 1 mmol/L sodium octanesulfonate and 0.1 mmol/L Na_2_EDTA : methanol, 98∶ 2 (v/v). Analyses were performed at 35°C at a flow rate of 0.7 mL/min. The electrochemical detector was set at +500 mV versus an Ag/AgCl reference electrode. A standard solution (10–20 µL) containing 500 ng each of 4-AHP (4-amino-3-hydroxyphenylalanine) and 3-AHP (3-amino-4-hydroxy- phenylalanine; 3-aminotyrosine; Sigma Ltd.) in 1 mL 0.1 M HCl was injected every 10 samples. The measures of PTCA and 4-AHP concentrations obtained by these methods have been shown to be reliable [66], [67].

In all statistical analyses we used PTCA and 4-AHP concentrations (µg/mg) as indexes of the concentrations of either eumelanin or, respectively, pheomelanin in the feather samples from the throat or belly feathers. We refrained from presenting estimates of eu- or pheomelanin concentrations obtained by back-converting PTCA and 4-AHP because back-conversion factors are not yet known with precision. Yet, we emphasize that because such conversion coefficients can be assumed to be constant between sexes and plumage regions, the present data are fully amenable to analysis of variation of either melanin form and of their relative concentration (Pheo:Eu ratio; see below) in relation to age, sex, as well as plumage region.

Throughout the text we refer to eu- and pheomelanins rather than to PTCA or, respectively, 4-AHP concentrations for clarity and easier reference.

### Heritability of Feather Coloration

Heritability of feather coloration was estimated using throat feathers collected from parents and their offspring when they were recruited as adult breeders. Feather samples for parents refer to the year when the offspring that was eventually recruited was generated. None of the recruits originated from the same brood. Because of very low, male-biased philopatry in our study population, in order to gather a sufficiently large sample we pooled the feather samples collected over 4 years and the analyses had to be restricted to male recruits only (see [68] for description of the original dataset). Because the feathers were collected in different years, coloration values were normalized to a within-year mean of 0 for offspring and male or female parents separately by subtracting the within-year mean value from each observation.

Because no pedigree was available, we estimated heritability using offspring-midparent regression, where heritability is estimated by the slope of the regression [69]. Such analysis incurs some potential bias. We used several analyses in order to try to rule out these biases. First, we exploited frequent occurrence of extra-pair offspring (i.e. nestlings sired by a male different from the social mate of the mother; see [53]) to estimate any environmental effect on parent-offspring resemblance due to sharing of the same environment during the nestling period. Paternity by the social father (i.e. whether the social father was also the biological father of the offspring or not) was assessed by genotyping both parents and the offspring at hypervariable microsatellite loci, as detailed in [53], [68], using small blood samples collected upon capture. Thus, we had two groups of male recruits (and families): those whose social father did not coincide with the biological father will be defined as extra-pair offspring (EPO), while the offspring whose social father was also their biological father will be qualified as biological offspring. Only families for which information for both parents was available could be included in the analyses. The slope of the EPO-social father regression of coloration variables should reflect the effect of common nesting environment (see also Discussion). Conversely, the slope of the regression between the biological offspring and the midparent phenotypic values should reflect heritability. Hence, non-significant EPO-social father regression would imply that any significant biological offspring-midparent relationship reflects heritable variation, and the slope of the biological parents-offspring regression can be used as an approximation of heritability. Second, because maternal effects are a likely cause of overestimation of heritability when using parent-offspring regression, we compared estimates from regression models using maternal phenotype only to the results from models using biological midparent values. If estimates from models using mother phenotypes are not higher than estimates from midparents or biological father models, then maternal effects can be excluded. Finally, the third assay was to compare the results from single biological parent regression to the offspring-midparent regression. The hypothesis being tested is that if resemblance among parent and offspring is due to additive genetic variance, offspring genotype is the average of its parents genotypes. In this case, the relationship between offspring and midparent phenotype should be stronger than the relationship between offspring phenotype and either of its parents.

### Statistical Analyses

To analyze variation in melanin concentration and color measures in relation to sex and age (yearlings vs. older individuals) we relied on linear models where the interaction between sex and age was removed if non-significant. The association between melanin concentration and color measures data was estimated using Pearson correlation coefficient. The α-level of statistical tests was always set at 0.05.

### Ethics Statement

Upon capture, barn swallows were kept in cloth bags in a safe position, as is standard practice in bird ringing studies. Overall, 300 individuals were sampled. Blood samples (<100 µl) were collected by puncturing the brachial vein. The puncturing site was accurately disinfected. All individuals were released as soon as possible, usually within 1 hour of capture. After being released, swallows behaved normally and observations at the nest on dozens of individuals confirmed that they resumed their normal breeding activities. The study was carried out under permission of the local authority (Regione Lombardia #M1.1997.00231 and #M1.2011.0002141) which is fully responsible responsible for authorizing animal studies in the wild, including any ethical issue related to the authorized protocol. No approval from an ethical committee was required for this study. Permission was obtained from the landowners to enter their property.

The data on which the present study is based can be obtained from NS upon request.

## Results

### Melanin Pigments in the Throat and Belly Feathers

**Figure 2:**
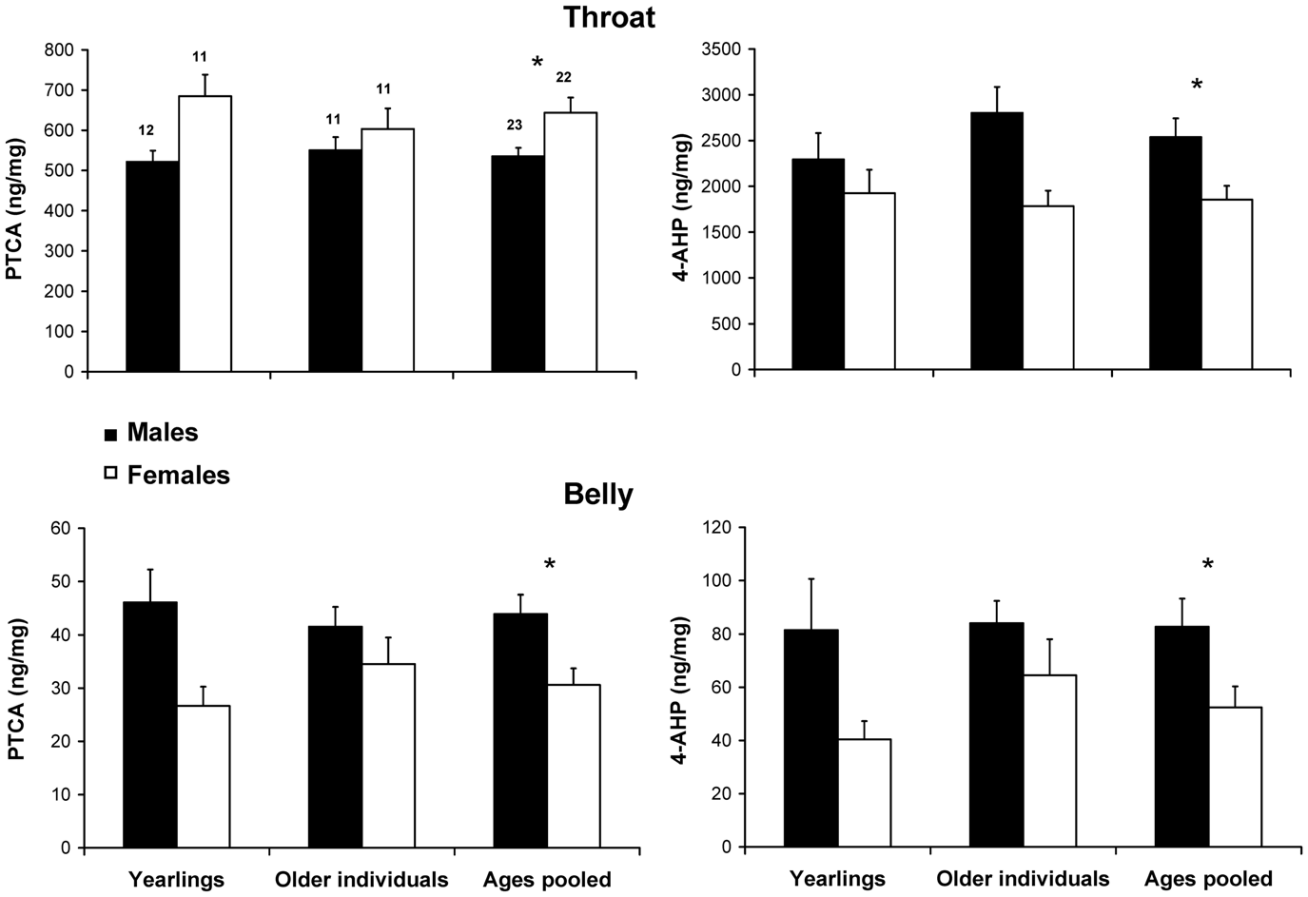
Mean (+ SE) concentration of eumelanin (PTCA) or pheomelanin (4-AHP) at two plumage regions of adult male and female barn swallows in two age classes (yearlings or older individuals). Because no age related variation existed, the sex-specific data pooled over the two age classes are also presented. Asterisks indicate a significant difference (P<0.05) between the sexes (see Table 1). Numbers in the body of the upper left figure are sample sizes of the sex by age classes.

The mean concentrations of PTCA and 4-AHP, which are proportional to absolute concentrations of eu- or, respectively, pheomelanin in throat and belly feathers are visualized in Figure 2 and age- and sex-related variation is analyzed in linear models in Table 1.

**Table 1:** Concentration of PTCA and 4-AHP, reflecting the concentrations of eu- and pheomelanin in two plumage regions of adult barn swallows in relation to sex and age (yearlings vs. older individuals).

		Eumelanin			Pheomelanin		
		F	df	P	F	df	P
Throat							
	Sex	6.57	1,42	0.014	7.12	1,42	0.011
	Age	0.34	1,42	0.562	0.54	1,42	0.467
	Sex×Age	1.71	1,41	0.198	1.63	1,41	0.210
Belly							
	Sex	7.61	1,42	0.009	5.34	1,42	0.026
	Age	0.10	1,42	0.755	0.98	1,42	0.329
	Sex×Age	1.68	1,41	0.203	0.65	1,41	0.425

The main effects of age and sex are estimated in a model excluding their non-significant interaction. See Figure 1 for sample sizes of the sex by age classes.

The concentration of both eu- and pheomelanins significantly differed between males and females and this was the case at both plumage regions (Table 1). However, in these models the concentration of melanins did not significantly vary according to age (Table 1; Fig. 2). In addition, the sex difference in melanin concentration did not significantly vary between yearlings and older individuals (Table 1; Fig. 2). Linear models with sex and plumage region as main effects showed that sex differences in the concentrations of both melanin forms varied between plumage regions (interaction between region and sex: eumelanin: F_1,86_ = 8.22, P = 0.005; pheomelanin: F_1,86_ = 6.48, P = 0.013). For both melanins, the concentration in males *relative* to females was larger in belly than in throat feathers (Fig. 2). Moreover, these models disclosed a significant main effect of plumage region (eumelanin: F_1,86_ = 674.80, P<0.001; pheomelanin: F_1,86_ = 276.46, P<0.001), with concentrations being higher in throat than in belly feathers for both pigments (Fig. 2).

The (log-transformed) Pheo:Eu ratio was significantly larger in males than in females in throat feathers (F_1,43_ = 10.93, P = 0.002) but did not differ between the sexes in belly feathers (F_1,43_ = 0.32, P = 0.577). As a result of the patterns of sex-related variation in melanin concentrations between plumage regions, there was a hint, though not statistically significant, to a sex by plumage region effect on log-transformed Pheo:Eu values (F_1,86_ = 2.96, P = 0.089). In this model, there were significant main effects of sex (F_1,86_ = 6.63, P = 0.012), with females having a larger ratio than males, and of plumage region (F_1,86_ = 52.54, P<0.001), with the proportion being significantly smaller in belly than in throat feathers (Fig. 2).

### Correlation of eu- and Pheomelanin Concentrations within and between Plumage Regions

**Figure 3:**
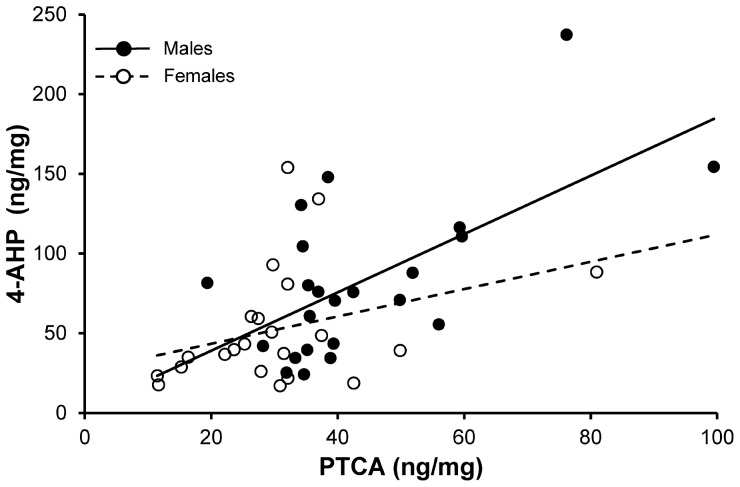
Relationship between concentration of pheomelanin (4-AHP) and eumelanin (PTCA) in the belly feathers of male and female adult barn swallows. The relationship was significant for males but not females (see Results). Linear regression lines are shown.

Within-sex there were no significant correlations among the concentrations of eu- and pheomelanin in either body region, with the only exception of belly feathers of males where the concentrations of the two melanin forms were strongly positively correlated (Table 2; Fig. 3). The corresponding correlation for females was also positive but statistically non-significant (Table 2; Fig. 3). The log-transformed Pheo:Eu values were also not correlated between plumage regions (males: r = 0.136, P = 0.535, females: r = 0.207, P = 0.356).

**Table 2:** Pearson correlation coefficients between PTCA and 4-AHP reflecting the concentrations of eu- and, respectively, pheomelanin in two plumage regions in either sex.

	Eumelanin, belly	Pheomelanin, throat	Pheomelanin, belly
Males			
Eumelanin, throat	0.101	−0.202	0.113
Eumelanin, belly		0.146	0.628**
Pheomelanin, throat			0.075
Females			
Eumelanin, throat	−0.228	−0.174	−0.205
Eumelanin, belly		0.248	0.339
Pheomelanin, throat			0.328

Sample size was 23 males and 22 females. ** indicates P = 0.0013; all the other correlation coefficients were non-significant (P>0.123).

### Tetrahedral Color Measures and Melanin Concentrations

**Figure 4:**
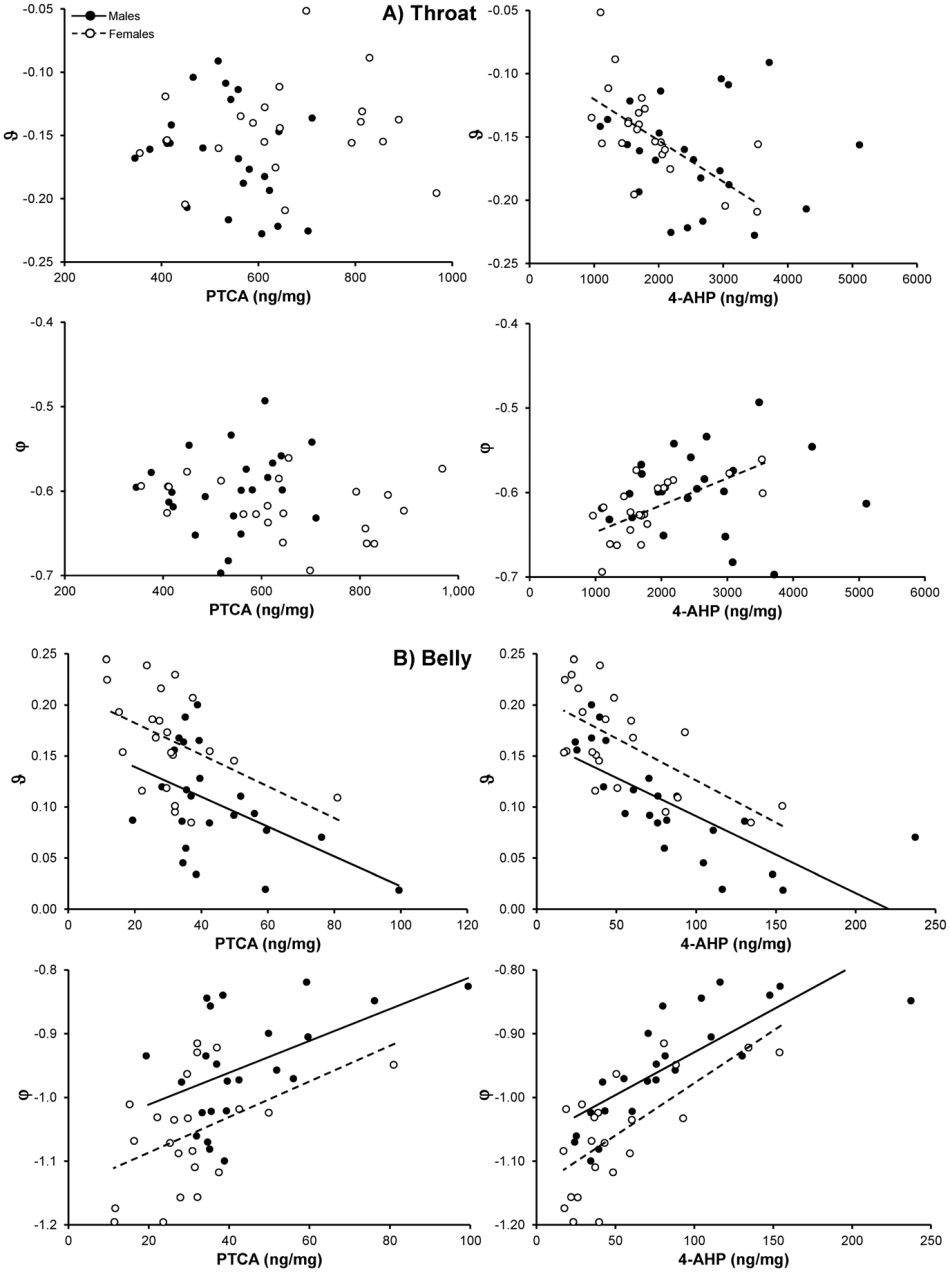
Relationships between ϑ and φ tetrahedral hue color components and eu- (PTCA) or pheomelanin (4-AHP). Panel A): throat feathers; panel B): belly feathers. Lines are fitted by linear regression to statistically significant relationships for either sex separately.

Within-sex there were poor associations between color components and melanin concentrations of throat feathers, with the exception of pheomelanin in females that was strongly associated with both the θ and φ color components (Table 3; Fig. 4a). In fact, a linear model disclosed a significant effect of the sex by pheomelanin concentration interaction on θ values (F_1,41_ = 4.15, P = 0.048) and a marginally non-significant effect on φ values (F_1,41_ = 3.58, P = 0.066), suggesting that the relationship between throat coloration and pheomelanin concentration differed between the sexes.

**Table 3:** Pearson correlation coefficients between the θ, φ (hue) or rA (saturation) tetrahedral color components and melanin concentration in throat or belly feathers.

	θ	φ	rA
Throat			
Eumelanin, males	−0.279	0.200	−0.171
Eumelanin, females	0.123	−0.253	0.033
Pheomelanin, males	−0.120	0.060	−0.308
Pheomelanin, females	−0.636**	0.650**	−0.404
Belly			
Eumelanin, males	−0.486*	0.503*	0.532**
Eumelanin, females	−0.466*	0.463*	0.360
Pheomelanin, males	−0.735**	0.791**	0.714**
Pheomelanin, females	−0.635**	0.694**	0.611**

Sample sizes are 23 males and 22 females. *: P<0.05; **: P<0.01.

The relationships between θ or φ hue color variables and eu- or pheomelanin concentration of belly feathers were invariably significant and consistent in sign between the sexes (Table 3). Moreover, the slope of the relationships did not differ between the sexes (P>0.498 in both analyses), rather being remarkably similar (Fig. 4b). In addition, color saturation (rA) was positively predicted by concentration of melanins in both sexes, though the association with eumelanin in females was not significant (Table 3).

A strikingly different pattern of association between color and the log-transformed Pheo:Eu values emerged among sexes and plumage regions. Both hue color components of throat feathers were significantly correlated with Pheo:Eu values in females but not in males (Table 4). Conversely, both hue color components and also saturation of belly feathers were significantly correlated with Pheo:Eu values in males but not in females (Table 4). The negative sign of the correlations between Pheo:Eu and the θ color component implies that feathers that looked paler were *relatively* more eu- than pheomelanized.

**Table 4:** Pearson correlation coefficients between the θ, φ (hue) or rA (saturation) tetrahedral color components and the (log-transformed) pheomelanin to eumelanin ratio.

	θ	φ	rA
Throat males	−0.030	0.004	−0.269
Throat females	−0.544**	0.639**	−0.319
Belly males	−0.689**	0.712**	0.512*
Belly females	−0.268	0.353	0.390

Sample sizes are 23 males and 22 females.

* P<0.05,

** P<0.01.

### Heritability of Feathers Coloration

**Figure 5:**
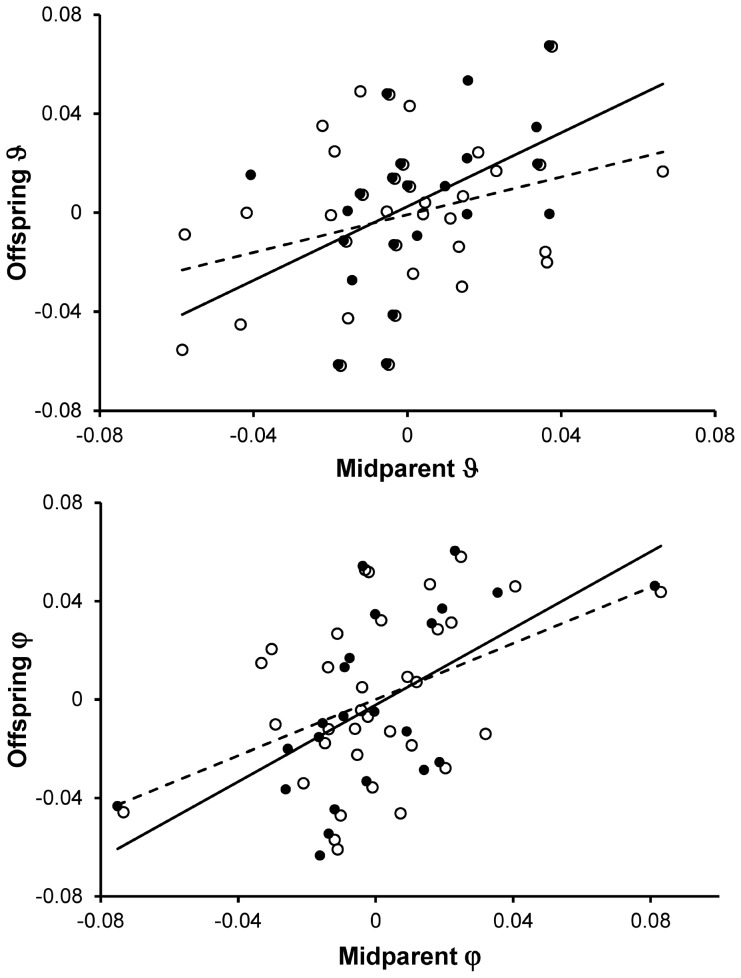
Relationship between offspring and the midparent ϑ and φ hue coloration components. Phenotypic values were normalized to a within-year mean of 0 to control for among-years sources of variation (see also Methods).

We had information on both parents and an offspring for 34 families: 23 with a biological offspring and 11 with an EPO. The slope of the relationship between biological offspring and midparent phenotypic values was significant for θ and φ but not for rA (Table 5; Fig. 5), giving heritability estimates of 0.81±0.28 and 0.80±0.23 for θ and φ respectively. Several lines of evidence suggest that what we detect is heritability. First, there were no significant relationships between EPO phenotypic values and the phenotypic value of the social father, although for rA the coefficient was surprisingly high (Table 5). Second, heritability estimates based on offspring-mother relation were not stronger than those from offspring-midparent relation, suggesting absence of detectable maternal effects. Finally, for θ, the offspring phenotype was more tightly related to the average of parents phenotype than to either of the biological parents, suggesting additivity. This was not the case for φ, which showed some stronger relation between biological father and son than between mother or midparent and offspring, suggesting some potential sex-specific effects.

**Table 5:** Linear regression coefficients (in parentheses: standard error; R^2^) of normalized (see Methods) phenotypic coloration values of offspring recorded when adults on normalized mid-parent phenotypic values, independently of the parental father also being the biological father (All offspring), or only in families where both social parents were also the biological parents (Biological offspring).

	θ	φ	rA
All offspring - Midparent	0.327 (0.205; 7.4)	0.570 (0.210; 18.7)*	0.325 (0.204; 7.4)
Biological offspring - Midparent	0.809 (0.285; 27.8)*	0.779 (0.226; 36.2)*	0.027 (0.261; 0.0)
Biological offspring - Mother	0.358 (0.190; 14.5)	0.365 (0.186; 15.5)	0.202 (0.188; 5.2)
Biological offspring - Father	0.286 (0.211; 8.1)	0.478 (0.185; 24.0)*	0.345 (0.224; 10.1)
Extra-pair offspring - Social father	−0.133 (0.220; 3.9)	−0.151 (0.297; 2.8)	0.295 (0.284; 10.7)

For biological offspring we also present heritability obtained from the regression between offspring an mothers or fathers (F). In line EPO the regression coefficient of the extra-pair offspring on social father phenotypic values is reported. Sample size for all offspring: 34; biological offspring: 23; EPO: 11. Heritability estimates for biological offspring – father (or mother) relationships can be obtained as 2×linear regression coefficient.

* significantly (P<0.05) larger than 0.

## Discussion

In a barn swallow population breeding in Italy we first showed that concentrations of eu- and pheomelanin in the throat and belly feathers differed between the sexes but did not vary with age and were larger in throat than in belly feathers. The relative concentrations of either melanin form also differed between males and females in throat but not in belly feathers, and the concentrations in males *relative* to females were larger in belly than in throat feathers. There were generally weak correlations between the concentrations of melanins within as well as among plumage regions in both sexes, with the only exception of belly feathers where the correlation between eu- and pheomelanin was positive, though statistically significant only in males. Coloration of belly feathers was strongly predicted by the concentration of both melanins whereas a correlation for throat feathers existed only with pheomelanin in females. In addition, the relative concentration of eu- and pheomelanin predicted coloration of throat feathers in females and that of belly feathers in males. Finally, high biological parents-offspring and low social father-extrapair offspring resemblance was observed in throat coloration, suggesting large heritability of color variation.

Concentration of melanins was estimated by chemical degradation of the melanin polymer and HPLC analysis of specific degradation products: PTCA (eumelanin) and 4-AHP (pheomelanin). Because back-conversion factors of degradation products to eu- and pheomelanin concentrations can be assumed to be consistent among sexes, age classes and plumage regions, the data we presented are fully amenable to analysis of sex- and plumage region-related variation of concentration of either melanin form. Sex-differences were inconsistent among plumage regions, as in throat feathers the sex-difference in eumelanin, but not in pheomelanin concentration was ‘reversed’ (i.e. females had larger concentration than males), and the Pheo:Eu values were consequently larger in males than females. Conversely, sex-differences were concordant for eu- or pheomelanin concentration in belly feathers. Hence, there appear to be different pigmentation strategies in either body region between males and females whose causes and function, if any, still remains to be elucidated. Sex differences in pigmentation are believed to be most often genetically based (see [3]), but the contribution of environmental effects cannot be dismissed a priori. Habitat quality effects potentially mediated by diet has been previously claimed to influence melanization in other species (e.g. [14], [70]–[72]). Moreover, an individual’s oxidative status can affect the course of melanin biosynthesis (see [73], [74]. In the barn swallow, timing of winter molt of the body feathers that we collected on the breeding grounds for pigment analyses may differ between the sexes. This is suggested by sex-related asynchrony of the post-breeding (pre-migratory) molt of body feathers [75], assuming that time schedules of the two molt episodes are correlated within individuals. Seasonal effects may thus influence melanogenesis because the environmental conditions to which either sex is exposed during molt of body feathers may differ. Because molt of body contour feathers proceeds in a postero-anterior direction [51], throat feathers are molted later than belly feathers. Seasonal variation in ecological conditions in the winter quarters of the Italian swallow population in Africa ([76]; our unpubl. results), with the rain season starting in late winter when molt is about to be completed, may thus partly contribute to sex-differences in melanization of throat feathers between males and females via sex-by-environment effects. Admittedly, the contribution of such interaction effects to sex differences in melanism is a mere speculation and the mechanisms that translate environmental effects into inter-individual or sex-related variation in melanism is unknown at present [77].

The concentrations of melanins were poorly correlated both within and among plumage regions. A significant correlation was only found between belly eu- and pheomelanin concentrations in males while the association was positive but far from statistical significance in females. The correlation between pheomelanin in either plumage region was also relatively large, though far from significance, only in females. Lack of significant association between pheo- and eumelanism in ‘mixed melanin’ pigmentation is consistent with previous findings [78] and suggests independent genetic and/or epigenetic control in biosynthesis pathways starting from common precursors. This pattern also suggests that no reciprocal constraint set by e.g. limitation of common precursors limits the production of either melanin form.

It should be noted that the amount of material available for analyses of melanins in throat feathers was very small. In addition, there is individual variation in size (length) of throat feathers as well as in the length and area of the brown terminal barbs (see also [34]), that were at the focus of our interest, and the grey/whitish basal barbs. Because trimming of exactly homologous portions of throat feathers from all individuals (and also from all the feathers belonging to the same individual) is technically difficult, we cannot exclude that the analyses of pigmentation and also those of coloration (see below) are partly blurred by random ‘noise’ in the data. Hence, the analyses including throat feathers pigmentation and coloration should be considered with this caveat in mind. Importantly, however, the present results were unaffected by potentially confounding effects of age. Age, in turn, did not significantly predict melanin concentration either per se or in combination with sex, suggesting that no age-related variation in melanization occurs in the barn swallow or that age effects arise only among very old, senescent individuals. Testing this possibility, however, would require ad hoc sampling of rare old individuals, which was beyond the main goals of the present study.

In belly feathers, both tetrahedral color hue components and also chroma were associated with the concentration of both melanin forms and consistently so in either sex. However, the relative amount of either melanin form in the belly feathers significantly predicted color only among males. In throat feathers, on the other hand, color was predicted only by pheomelanin and the pheo- to eumelanin concentration ratio in females. The reason for such discrepancies between plumage regions and sexes is matter of speculation. Practical difficulties in reflectance recordings in throat feathers (see also above) or great excess of pheo- compared to eumelanin cannot explain why a strong relationship between pheomelanin concentration and color was found in females but not males. It could be speculated that structural differences in the feather barbs occur between the sexes [34].

Pigmentation of belly feathers and its association with color has been previously analyzed in a barn swallow population of the subspecies *H. r. erythrogaster* from North Eastern USA [34]. As the ancient Greek ethimology of the subspecies’ scientific name itself suggests (□ρυθρός = red; γαστήρ = belly), American swallows have more markedly brownish belly plumage. Some of the findings were similar in the present and in the previous study by McGraw et al. [34]. In both studies, coloration (though quantified by different approaches) was predicted by concentration of both melanin forms, and a significant association between coloration variables and the relative amount of either melanin form was recorded for males but not females. On the other hand, there are also striking differences between the results of the two studies. First, a sex difference in concentration of both melanins was found in this study whereas in *H. r. erythrogaster* it was recorded only for pheomelanin. Second, a significant (and positive) correlation between pheo- and eumelanin concentrations in the present study was recorded only among males whereas it was recorded only among females in *H. r. erythrogaster*. No analyses on throat feathers that could serve as a basis for comparison were apparently done on American barn swallows. Interestingly, however, there are also marked differences in sex-related melanic profiles of throat feathers between the swallows we studied in Italy and those from a relatively small sample of *H. r. rustica* breeding individuals from Scotland [79]: while we found significant differences in the concentrations of both melanin forms between the sexes, with eumelanin being more concentrated in females and pheomelanin being more concentrated in males, no clear difference existed between the sexes in Scotland, as can be judged from the extensive overlap between confidence intervals of the reported mean concentrations [79]. The striking differences in the pigmentation of throat or belly feathers between barns wallows from northern Italy and those from Scotland or *H. r. erythorgaster* from North America may suggest that pigmentation has undergone genetic divergence between these populations, although the possibility of non-negligible environmental variance cannot be ruled out. Thus, given what is known about the genetic and physiological machinery behind pheo- and eumelanogenesis and what has been hypothesized about the role of melanin in communication, these results suggest that any socio-sexual role of melanin-based coloration could also differ between geographical populations. This would of course not be surprising given ample evidence for geographical variation in natural and sexual selection regimes among geographical populations ([80]; see [81] and references therein).

Color measurements in the present study were obtained while taking the spectral sensitivity profile of passerines into account, and that all the significant associations of pigmentation variables that were recorded with the θ (‘visible’) color component were also recorded (though reversed in sign) with the φ (UV) color component. Thus, melanin pigmentation appears to covary with pigmentation both in the ‘visible’ and UV bands, which can therefore both serve as mediators of any signal that melanin-based coloration may convey. Coloration in the UV band should therefore be taken into account in future studies of coloration. Unfortunately, however, no information is available on the structure of the feathers barbs that may influence reflectance in the ‘visible’ or in the UV coloration in the barn swallow.

Finally, low estimates of social father-extrapair offspring resemblance in throat hue color components suggest that the large biological parents-offspring resemblance in hue reflects genetic effects and that coloration thus has large heritability. We controlled and tested for potentially confounding effects such as year and maternal effects and still found high values of heritability. Heritability of melanin-based coloration may be generally high and has been shown to be similar or even larger than that suggested by the present slopes of the biological parents-offspring regressions in some instances (e.g. h^2^ = 0.81 for mainly pheomelanin-based coloration in the barn owl (*Tyto alba*) [37]; h^2^ = 0.93 in the tawny owls (*Strix aluco*) [78]). Moreover, coloration has been claimed to be heritable also in the East-Mediterranean barn swallow subspecies (*H. r. transitivia*) (Y. Vortman unpublished data cited in [82]). Compared to heritability estimates generally found in other traits (around 0.3; [83], [84]), these values are thus extremely high.

These high heritability values suggest that some common environmental effects could be biasing heritability upwards. For example, throat feathers are molted in Africa and parents and offspring may tend to share the same wintering quarters and molt at the same time of the year, so that the possibility that biological parents-offspring resemblance estimates were inflated by common environment effects cannot be ruled out. Unfortunately, it should be noted that controlling for such effects in studies of this and other migratory species with similar molt strategies will hardly be possible because of obvious practical constraints on the study of molt of specific individuals of known pedigree in Africa.

In conclusion, we identified the melanin determinants of coloration of throat and belly feathers of barn swallows breeding in Italy. Our results indicate extensive differences between the sexes in pigmentation and thus in coloration both within and among plumage regions even in a species with no ‘discrete’ sexual dichromatism (i.e. where homologous plumage regions have similar coloration in either sex), but no age-related variation in pigmentation, at least among the most common age classes in the population. Meanwhile, our results also show that extensive differences exist in several aspects of pigmentation and its relationship with coloration among geographical populations. Finally, by exploiting natural levels of extra-pair paternity we uncovered high heritability of coloration. Further studies are thus needed to identify the function(s) of coloration in our study population and its patterns of covariation with life-history traits.

## References

[1] Majerus MEN (1998) Melanism, Evolution in Action. Oxford: Oxford University Press.

[2] Jawor JM, Breitwisch R (2003) Melanin ornaments, honesty, and sexual selection. Auk 120: 249–265.

[3] Roulin A (2004) The evolution, maintenance and adaptive function of genetic colour polymorphism in birds. Biol Rev 79: 815–848. 15682872 10.1017/s1464793104006487

[4] Hill GE, McGraw KJ (2006a) Bird Coloration. Volume II. Function and Evolution. Cambridge: Harvard University Press.

[5] Hoekstra HE (2006) Genetics, development and evolution of adaptive pigmentation in vertebrates. Heredity 97: 222–234. 16823403 10.1038/sj.hdy.6800861

[6] Johannesson K, Ekendahl A (2002) Selective predation favouring cryptic individuals of marine snails (*Littorina*). Biol J Linn Soc 76: 137–144.

[7] Roulin A, Wink M (2004) Predator-prey polymorphism: relationships and the evolution of colour a comparative analysis in diurnal raptors. Biol J Linn Soc 81: 565–578.

[8] Hoekstra HE, Krenz JG, Nachman MW (2005) Local adaptation in the rock pocket mouse (*Chaetodipus intermedius*): natural selection and phylogenetic history of populations. Heredity 94: 217–228. 15523507 10.1038/sj.hdy.6800600

[9] Singaravelan N, Pavlicek T, Beharav A, Wakamatsu K, Ito S, et al (2010) Spiny mice modulate eumelanin to pheomelanin ratio to achieve cryptic coloration in “Evolution Canyon”. Israel. PLoS ONE 5: e–8708. 10.1371/journal.pone.0008708 PMC2806840 20090935

[10] Badyaev AV, Hill GE (2000) Evolution of sexual dichromatism: contribution of carotenoid versus melanin-based coloration. Biol J Linn Soc 69: 153–172.

[11] Møller AP, Biard C, Blount JD, Houston DC, Ninni P, et al (2000) Carotenoid-dependent signals: indicators of foraging efficiency, immunocompetence, or detoxification ability? Avian Poult Biol Rev 11: 137–159.

[12] von Schantz T, Bensch S, Grahn M, Hasselquist D, Wittzell H (1999) Good genes, oxidative stress and condition-dependent sexual signals. Proc R Soc Lond B 266: 1–12. 10.1098/rspb.1999.0597 PMC1689644 10081154

[13] Fitze PS, Richner H (2002) Differential effects of a parasite on ornamental structures based on melanins and carotenoids. Behav Ecol 13: 401–407.

[14] Fargallo JA, Laaksonen T, Korpimäki E, Wakamatsu K (2007) A melanin-based trait reflects environmental growth conditions of nestling male Eurasian kestrels. Evol Ecol 21: 157–171.

[15] Roulin A, Almasi B, Rossi-Pedruzzi A, Ducrest A-L, Wakamatsu K, et al (2008) Corticosterone mediates the condition-dependent component of melanin-based coloration. Anim Behav 75: 1351–1358.

[16] Siefferman L, Hill GE (2005) Evidence for sexual selection on strucutral plumage coloration in female Eastern Bluebirds (*Sialia sialis*). Evolution 59: 1819–1828. 16331840

[17] Costantini D, Møller AP (2008) Carotenoids are minor antioxidants in birds. Funct Ecol 22: 367–370.

[18] Fujii R (2000) The regulation of motile activity of fish chromatophores. Pigment Cell Res 13: 300–319. 11041206 10.1034/j.1600-0749.2000.130502.x

[19] Mathger LM, Denton EJ, Marshall NJ, Hanlon NT (2009) Mechanisms and behavioural functions of structural coloration in cephalopods. J Roy Soc Interf 6: S149–S163. 10.1098/rsif.2008.0366.focus PMC2706477 19091688

[20] Meunier J, Figueuredo Pinto S, Burri R, Roulin A (2011) Eumelanin-based coloration and fitness parameters in birds: a meta-analysis. Behav Ecol Sociobiol 65: 559–567.

[21] Prota G (1992) Melanins and Melanogenesis. New York: Academic Press. 1–290.

[22] Hearing VJ (1998) The regulation of melanin production. In: Nordlund JJ, Boissy R, Hearing VJ, King RA, Ortonne J-P eds. The Pigmentary System. Physiology and Pathophysiology. New York: Oxford University Press. 423–438.

[23] Ito S, Wakamatsu K, Ozeki H (2000) Chemical analysis of melanins and its application to the study of the regulation of melanogenesis. Pigment Cell Res 13: 103–109. 11041366 10.1034/j.1600-0749.13.s8.19.x

[24] Wakamatsu K, Ito S, Rees JL (2002) The usefulness of 4-amino-3-hydroxyphenylalanine as a specific marker of pheomelanin. Pigment Cell Res 15: 225–232. 12028587 10.1034/j.1600-0749.2002.02009.x

[25] Simon JD, Peles D, Wakamatsu K, Ito S (2009) Current challenges in understanding melanogenesis: bridging chemistry, biological control, morphology, and function. Pigment Cell Mel Res 22: 563–579. 10.1111/j.1755-148X.2009.00610.x 19627559

[26] Haase E, Ito S, Sell A, Wakamatsu K (1992) Melanin concentrations in feathers from wild and domestic pigeons. J Hered 83: 64–67.

[27] Haase E, Ito S, Wakamatsu K (1995) Influences of sex, castration, and androgens on the eumelanin and pheomelanin contents of different feathers in wild mallards. Pigment Cell Res 8: 164–170. 7567793 10.1111/j.1600-0749.1995.tb00658.x

[28] Ito S, Wakamatsu K (2011) Human hair melanins: what we have learned and have not learned from mouse coat color pigmentation. Pigm Cell Mel Res 24: 63–74. 10.1111/j.1755-148X.2010.00755.x 20726950

[29] Dreiss AN, Roulin A (2010) Age-related change in melanin-based coloration of Barn owls (*Tyto alba*): females that become more female-like and males that become more female-like perform better. Biol J Linn Soc 101: 689–704.

[30] Commo S, Wakamatsu K, Lozano I, Panhard S, Loussouarn G, et al (2012) Age-dependent changes in eumelanin composition in hairs of various ethnic origin. Int J Cosm Sci 34: 102–107. 10.1111/j.1468-2494.2011.00691.x 22017184

[31] Ducrest A-L, Keller L, Roulin A (2008) Pleiotropy and the melanocortin system, coloration and behavioural syndromes. Trends Ecol Evol 23: 502–510. 18644658 10.1016/j.tree.2008.06.001

[32] Roulin A, Almasi B, Jenni L (2010a) Temporal variation in glucocorticoid levels during the resting phase os associated in opposite way with maternal and paternal melanic coloration. J Evol Biol 23: 2046–2053. 20840305 10.1111/j.1420-9101.2010.02086.x

[33] Hill GE, McGraw KJ (2006b) Bird Coloration. Volume I. Mechanisms and Measurements. Cambridge: Harvard University Press.

[34] McGraw KJ, Safran RJ, Wakamatsu K (2005) How feather colour reflects its melanin content. Funct Ecol 19: 816–821.

[35] Galván I, Møller AP (2009) Different roles of natural and sexual selection on senescence of plumage colour in the barn swallow. Funct Ecol 23: 302–309.

[36] Griffith SC, Owens IPF, Burke T (1999) Environmental determination of a sexually selected trait. Nature 400: 358–360.

[37] Roulin A, Dijkstra C (2003) Genetic and environmental components of variation in eumelanin and phaeomelanin sex-traits in the barn owl. Heredity 90: 359–364. 12714980 10.1038/sj.hdy.6800260

[38] Bize P, Gasparini J, Klopfenstein A, Altwegg R, Roulin A (2006) Melanin-based colouration is a non-directionally selected sex-specific signal of offspring development in the Alpine swift. Evolution 60: 2370–2380. 17236427

[39] Jensen H, Svorkmo-Lundberg T, Ringsby TH, Sæther BE (2006) Environmental influence and cohort effects in a sexual ornament in the house sparrow, *Passer domesticus* . Oikos 114: 212–224.

[40] Roulin A, Altwegg R, Jensen H, Steinsland I, Schaub M (2010b) Sex-dependent selection on autosomal melanic female ornament promotes the evolution of sex ratio bias. Ecol Lett 13: 616–626. 20337696 10.1111/j.1461-0248.2010.01459.x

[41] Hearing VJ, Tsukamoto K (1991) Enzymatic control of pigmentation in mammals. FASEB J 5: 2902–2909. 1752358

[42] Norris K (1993) Heritable variation in a plumage indicator of viability in male great tits Parus major. Nature 362: 537–539.

[43] McKenzie CA, Eakamatsu K, Hanchard NA, Forrester T, Ito S (2007) Childhood malnutrition is associated with a reduction in the total melanin content of scalp. Br J Nutr 98: 159–164. 17381963 10.1017/S0007114507694458

[44] Adachi K, Wakamatsu K, Ito S, Matsubara H, Nomura K, et al (2010) A close relationship between androgen levels and eumalnogenesis in the teleost red seabream (*Pagrus major*): quantitative analysis of its seasonal variation and effects of oral treatment with methyl-testosterone. Comp Biochem Physiol 156: 184–189. 10.1016/j.cbpa.2010.01.021 20138232

[45] Safran RJ, McGraw KJ (2004) Plumage coloration, not the length or symmetry of tail streamers, is a sexually selected trait in North American barn swallows. Behav Ecol 15: 455–461.

[46] Endler JA, Mielke PW Jr (2005) Comparing entire coilour patterns as birds see them. Biol J Linn Soc 86: 405–431.

[47] Stoddard MS, Prum RO (2008) Evolution of avian plumage color in a tetrahedral color space: a phylogenetic analysis of new world buntings. Am Nat 171: 755–776. 18419340 10.1086/587526

[48] Pike TW, Bjerkeng B, Blount JD, Lindström J, Metcalfe NB (2011) How integument colour reflects its carotenoid content: a stickleback’s perspective. Funct Ecol 25: 297–304.

[49] Møller AP (1994) Sexual selection and the barn swallow. Oxford: Oxford University Press.

[50] Cramp S (1998) The complete birds of the western Palearctic on CD-ROM. Oxford: Oxford University Press.

[51] Turner A (2006) The barn swallow. London: T & AD Poyser.

[52] Ginn HB, Melville DS (1983) Moult in Birds. Norfolk: British Trust for Ornithology.

[53] Saino N, Primmer CR, Ellegren H, Møller AP (1997) An experimental study of paternity and tail ornamentation in the barn swallow (*Hirundo rustica*). Evolution 51: 562–570. 28565341 10.1111/j.1558-5646.1997.tb02443.x

[54] Safran RJ, Neuman CR, McGraw KJ, Lovette IJ (2005) Dynamic paternity allocation as a function of plumage color in barn swallows. Science 309: 2210–2212. 16195460 10.1126/science.1115090

[55] Møller AP, Saino N, Taramino G, Galeotti P, Ferrario S (1998) Paternity and multiple signaling: effects of a secondary sexual character and song on paternity in the barn swallow. Am Nat 151: 236–242. 18811354 10.1086/286114

[56] Eikenaar C, Whitham M, Komdeur J, van der Velde M, Moore IT (2011) Testosterone, plumage colouration and extra-pair paternity in male North American barn swallows. PLoS ONE 6: e23288. 21853105 10.1371/journal.pone.0023288 PMC3154291

[57] Vortman Y, Lotem A, Dor R, Lovette IJ, Safran RJ (2011) The sexual signals of the East-Mediterranean barn swallow: a different swallow tale. Behav Ecol 22: 1344–1352.

[58] Saino N, Martinelli R, Romano M (2008) Ecological and phenological covariates of offspring sex ratio in barn swallows. Evol Ecol 22: 659–674.

[59] Saino N, Calza S, Ninni P, Møller AP (1999) Barn swallows trade survival against offspring condition and immunocompetence. J Anim Ecol 68: 999–1009.

[60] Saino N, Caprioli M, Romano M, Boncoraglio G, Rubolini D, et al (2011) Antioxidant defences predict long-term survival in a passerine bird. PlosOne 6: e19593. 10.1371/journal.pone.0019593 PMC3089629 21573124

[61] Saino N, Romano M, Ambrosini R, Rubolini D, Boncoraglio G, et al (2012) Longevity and lifetime reproductive success of barn swallow offspring are predicted by their hatching date and phenotypic quality. J Anim Ecol 81: 1004–1012. 22531043 10.1111/j.1365-2656.2012.01989.x

[62] Goldsmith TH (1990) Optimization, constraint, and history in the evolution of eyes. Q Rev Biol 65: 281–322. 2146698 10.1086/416840

[63] Antonov A, Stokke BG, Vikan JR, Fossoy F, Ranke PS, et al (2010) Egg phenotype differentiation in sympatric cuckoo *Cuculus canours* gentes. J Evol Biol 23: 1170–1182. 20345810 10.1111/j.1420-9101.2010.01982.x

[64] Lessells CM, Boag PT (1987) Unrepeatable repeatabilities: a common mistake. Auk 104: 116–121.

[65] Ito S, Fujita K (1985) Microanalysis of eumelanin and pheomelanin in hair and melanomas by chemical degradation and liquid chromatography. Anal Biochem 144: 527–536. 3993914 10.1016/0003-2697(85)90150-2

[66] Ito S, Nakanishi Y, Valenzuela RK, Brilliant MH, Kolbe L, et al (2011) Usefulness of alkaline hydrogen peroxide oxidation to analyze eumelanin and pheomelanin in various tissue samples: application to chemical analysis of human hair melanins. Pigment Cell Mel Res 24: 605–613. 10.1111/j.1755-148X.2011.00864.x 21535429

[67] Wakamatsu K, Ito S, Rees JL (2002) The usefulness of 4-amino-3-hydroxyphenylalanine as a specific marker of pheomelanin. Pigment Cell Res 15: 225–232. 12028587 10.1034/j.1600-0749.2002.02009.x

[68] Saino N, Martinelli R, Romano M, Møller AP (2003) High heritable variation of a male secondary sexual character revealed by extra-pair fertilization in the barn swallow. Ital J Zool 70: 167–174.

[69] Lynch M, Walsh B (1998) Genetics and analysis of quantitative traits. Sunderland: Sinauer.

[70] Griffith SC (2000) A trade-off between reproduction and a condition-dependent sexually selected ornament in the house sparrow *Passer domesticus* . Proc R Soc Lond B 267: 1115–1119. 10.1098/rspb.2000.1116 PMC1690642 10885516

[71] Niecke M, Rothlaender S, Roulin A (2003) Why do melanin ornaments signal individual quality? Insights from metal element analysis of barn owl feathers. Oecologia 137: 153–158. 12811535 10.1007/s00442-003-1307-3

[72] Poston JP, Hasselquist D, Stewart IRK, Westneat DF (2005) Dietary amino acids influence plumage traits and immune responses of male house sparrows, *Passer domesticus*, but not as expected. Anim Behav 70: 1171–1181.

[73] Galván I, Alonso-Alvarez C (2008) An intracellular antioxidant determines the expression of a melanin-based signal in a bird. PLoS ONE 3: e3335. 18833330 10.1371/journal.pone.0003335 PMC2556083

[74] Roulin A, Almasi B, Meichtry-Stier KS, Jenni L (2011) Eumelanin- and pheomelanin-based colour advertise resistance to stress in opposite ways. J Evol Biol 24: 2241–2247. 21745253 10.1111/j.1420-9101.2011.02353.x

[75] Rubolini D, Massi A, Spina F (2002) Replacement of body feathers is associated with low premigratory energy stores in a long-distance migratory bird, the barn swallow. J Zool 258: 441–447.

[76] Saino N, Szèp T, Romano M, Rubolini D, Møller AP (2004) Ecological conditions during winter predict arrival date at the breeding quarters in a trans-Saharan migratory bird. Ecol Lett 7: 21–25.

[77] Griffith SC, Parker TH, Olson VA (2006) Melanin- versus carotenoid-based sexual signals: is the difference really so black and red? Anim Behav 71: 749–763.

[78] Gasparini J, Bize P, Piault R, Wakamatsu K, Blount JD, et al (2009) Strength and cost of an induced immune response are associated with a heritable melanin-based colour trait in female tawny owls. J Anim Ecol 78: 608–616. 19175442 10.1111/j.1365-2656.2008.01521.x

[79] McGraw KJ, Safran RJ, Evans MR, Wakamatsu K (2004) European barn swallows use melanin pigments to color their feathers brown. Behav Ecol 5: 889–891.

[80] Møller AP, Chabi Y, Cuervo JJ, de Lope F, Kilpimaa J, et al (2006) An analysis of continent-wide patterns of sexual selection in a passerine bird. Evolution 60: 856–868. 16739465

[81] Aparicio JM, Muñoz A, Bonal R, Møller AP (2012) Population differences in desnity and resource allocation of ornamental tail feathers in the barn swallow. Biol J Linn Soc 105: 925–936.

[82] Dor R, Safran RJ, Vortman Y, Lotem A, McGowan A, et al (2012) Population genetics and morphological comparisons of migratory European (*Hirundo rustica rustica*) and sedentary East-Mediterranean (*Hirundo rustica transitiva*) barn swallows. Heredity 103: 55–63. 10.1093/jhered/esr114 22071313

[83] Houle D (1992) Comparing evolvability and variability of quantitative traits. Genetics 130: 195–204. 1732160 10.1093/genetics/130.1.195 PMC1204793

[84] Hansen TF, Pelabon C, Houle D (2011) Heritability is not Evolvability. Evol Biol 38: 258–277.

